# Identification of Potential Key Genes in the Pathogenesis of Chronic Obstructive Pulmonary Disease Through Bioinformatics Analysis

**DOI:** 10.3389/fgene.2021.754569

**Published:** 2021-11-03

**Authors:** Qingzhou Guan, Yange Tian, Zhenzhen Zhang, Lanxi Zhang, Peng Zhao, Jiansheng Li

**Affiliations:** ^1^ Academy of Chinese Medical Sciences, Henan University of Chinese Medicine, Zhengzhou, China; ^2^ Henan Key Laboratory of Chinese Medicine for Respiratory Disease, Collaborative Innovation Center for Chinese Medicine and Respiratory Diseases Co-constructed by Henan Province and Education Ministry of P.R. China, Henan University of Chinese Medicine, Zhengzhou, China; ^3^ Department of Respiratory Diseases, The First Affiliated Hospital of Henan University of Chinese Medicine, Zhengzhou, China

**Keywords:** chronic obstructive pulmonary disease, gene expression, differentially expressed genes, protein–protein interaction network, ceRNA network

## Abstract

Chronic obstructive pulmonary disease (COPD) is a common respiratory disease with high morbidity and mortality. The etiology of COPD is complex, and the pathogenesis mechanisms remain unclear. In this study, we used rat and human COPD gene expression data from our laboratory and the Gene Expression Omnibus (GEO) database to identify differentially expressed genes (DEGs) between individuals with COPD and healthy individuals. Then, protein–protein interaction (PPI) networks were constructed, and hub genes were identified. Cytoscape was used to construct the co-expressed network and competitive endogenous RNA (ceRNA) networks. A total of 198 DEGs were identified, and a PPI network with 144 nodes and 355 edges was constructed. Twelve hub genes were identified by the cytoHubba plugin in Cytoscape. Of these genes, *CCR3*, *CCL2*, *COL4A2*, *VWF*, *IL1RN*, *IL2RA*, and *CCL13* were related to inflammation or immunity, or tissue-specific expression in lung tissue, and their messenger RNA (mRNA) levels were validated by qRT-PCR. *COL4A2*, *VWF*, and *IL1RN* were further verified by the GEO dataset GSE76925, and the ceRNA network was constructed with Cytoscape. These three genes were consistent with COPD rat model data compared with control data, and their dysregulation direction was reversed when the COPD rat model was treated with effective-component compatibility of Bufei Yishen formula III. This bioinformatics analysis strategy may be useful for elucidating novel mechanisms underlying COPD. We pinpointed three key genes that may play a role in COPD pathogenesis and therapy, which deserved to be further studied.

## Introduction

Chronic obstructive pulmonary disease (COPD) is a common chronic respiratory disease, characterized by airflow limitation that is not completely reversible. COPD patients experience declines in lung function resulting in great economic and social burdens worldwide (Vestbo et al., 2013; Ma et al., 2019; Mao et al., 2019; Sun et al., 2019; Chen et al., 2020). Studies reveal that COPD will become the third leading cause of death worldwide by 2030, and it is estimated that over 5.4 million people will die from COPD annually from 2060 (Singh et al., 2019; Belchamber and Donnelly, 2020; Chen et al., 2020).

Before 2011, COPD was primarily evaluated based on forced expiratory volume during the first second (FEV1). Patients were divided according to the Global Initiative for Chronic Obstructive Pulmonary Disease (GOLD) stages (GOLD 1, mild; GOLD 2, moderate; GOLD 3, severe; and GOLD 4, very severe). However, FEV1 is only a partial descriptor of this multisystemic disease and cannot reflect its complexity and effects beyond pulmonary function, including reductions in health status and physical performance (Bernabeu-Mora et al., 2020). In 2011, GOLD provided a multidimensional evaluation method that combined the FEV1 value with an individual’s previous exacerbations and symptoms history, including dyspnea based on the modified Medical Research Council (mMRC) scale and health status according to the Chronic obstructive pulmonary disease Assessment Test (CAT) (Bernabeu-Mora et al., 2020). The GOLD 2011 assessment classified COPD patients into four groups (A–D): Group A, patients with low risk and few symptoms; Group B, patients with low risk and many symptoms; Group C, patients with high risk and few symptoms; and Group D, patients with high risk and many symptoms. The detailed classification criteria are shown in Vestbo et al. (2013).

COPD is a multifactorial disease with complex interaction between genes and the environment. Cigarette smoking is a major risk factor for disease development (Zhang and Xu, 2020). When exposed to cigarette smoke, lung macrophages, accounting for approximately 80%–90% of the immune cell population, play a vital role in processing and clearing particles from the lungs (Yamasaki and Eeden, 2018; Eapen et al., 2019). Continuous exposure to cigarette smoke markedly depletes intracellular antioxidants, such as glutathione, causing excessive oxidative stress that suppresses the lung macrophages’ bacterial phagocytic and efferocytic functions (Donnelly and Barnes, 2012). Moreover, lung macrophages in COPD generate a pro-inflammatory scenario that may cause tissue damage, and defective immune surveillance and protective (phagocytic) functions that collectively contribute to the progression of COPD (Yamasaki and Eeden, 2018; Eapen et al., 2019). Other risk factors, such as age, sex, and socioeconomic status, are also involved in COPD development (Gershon et al., 2011; Landis et al., 2014; Beran et al., 2015; Mercado et al., 2015). Fewer than 50% of heavy smokers develop COPD (Rennard and Vestbo, 2006; Wang et al., 2020), indicating that genetics may play a key role in COPD pathogenesis (Rennard and Vestbo, 2006). For example, alpha-1 antitrypsin deficiency (AATD), a major circulating inhibitor of serine proteases, is a well-documented genetic risk factor that predisposes an individual to COPD (Stoller and Aboussouan, 2005). Moreover, the gene encoding matrix metalloproteinase 12 (MMP-12) and glutathione S-transferase are reportedly linked to a decline in lung function or risk of COPD (Hunninghake et al., 2009; Ding et al., 2019). Researchers have reported some genetic loci associated with COPD (FEV1 or FEV1/FVC as the phenotype) including markers near the alpha-nicotinic acetylcholine receptor, *HHIP*, *GPR126*, *ADAM19*, *AGER-PPT2*, *FAM13A*, *PTCH1*, *PID1*, and HTR4 (Pillai et al., 2009; Cho et al., 2010; Hancock et al., 2010; Repapi et al., 2010; Soler Artigas et al., 2011; Cho et al., 2014). The 15q24/25 locus in nAChR is correlated with the occurrence and progression of emphysema, suggesting that it is causally involved in alveolar destruction, a pathogenic mechanism potentially shared with COPD (Lambrechts et al., 2010). In addition, the precise pathogenesis mechanisms of COPD remain unclear (Chen et al., 2020; Wang et al., 2020). Thus, it is critical to elucidate the molecular mechanisms underlying COPD for its treatment.

Transcriptomic and high-throughput microarray/RNA-seq analyses have been widely applied to many diseases, including COPD, to explore the mechanisms and advance diagnosis/treatment (Kaczkowski et al., 2016; Macias-Segura et al., 2018; Demircioglu et al., 2019; Li et al., 2019; Morrow et al., 2019; Carr et al., 2020; Cheng et al., 2021). In addition, the competitive endogenous RNA (ceRNA) networks can also provide new insights into the mechanisms of disease development in a transcriptional regulatory network (Salmena et al., 2011). By combining high-throughput microarray/RNA-seq data and bioinformatics analysis algorithms, we can potentially identify key genes closely related to the occurrence of diseases and provide guidance for treatment.

In this study, we applied human COPD gene expression data from the Gene Expression Omnibus (GEO) database and rat data from our laboratory. First, we identified consistent differentially expressed genes (DEGs) among human and rat data. The protein–protein interaction (PPI) network was constructed using the STRING database (https://www.string-db.org/), and Cytoscape software was applied to identify cluster modules related to COPD. Five algorithms (Degree, MCC, MNC, DMNC, and Clustering Coefficient) were used to identify hub genes in the cytoHubba plugin within Cytoscape. Based on the online databases BioGPS and GeneCards, those genes were further optimized. Then, target microRNAs (miRNAs) of the identified genes were predicted by four online miRNA databases, and the gene–miRNA target network was constructed with Cytoscape. Subsequently, we further validated/optimized the identified genes using a GEO dataset and COPD rat data, and ceRNA networks were constructed based on the prediction results of long noncoding RNAs (lncRNAs) using starBase database. We also found the genes that were reversed when the COPD rats were treated with effective-component compatibility of Bufei Yishen formula III (ECC-BYF III). This work provides insight into the mechanisms of disease development in COPD at the transcriptome level, contributing potential guidance for COPD treatment.

## Methods

### Data Acquisition and Identification of Differentially Expressed Genes

Multiple gene expression profiles of human COPD and normal lung tissue samples were downloaded from the GEO database, as shown in Table 1. For the data detected by the Affymetrix platform, the raw messenger RNA (mRNA) expression data (.CEL files) were downloaded, and the Robust Multi-array Average (RMA) algorithm was applied for background adjustment. Processed data detected by the Illumina or Agilent platform were directly downloaded. The probe ID was mapped to Entrez Gene ID with the corresponding platform file for the array-based data, while for the seq-based data, gene symbols were mapped to the Entrez Gene ID with the bioDBnet database. The scarcity of subtype information for the COPD data meant we directly analyzed the COPD samples in contrast with control samples, regardless of the COPD subtype. Thus, the potential genes identified in this study are general. As COPD data accumulate and more clinical information is gathered, subtype information should be considered for further study.

**TABLE 1:** Human chronic obstructive pulmonary disease (COPD) data used in this study.

	Platform	Normal	COPD
GSE38974	Agilent GPL4133	9	23
GSE8581	Affymetrix GPL570	19	16
GSE57148^a^	Illumina GPL11154	91	98

a Note. The RNA_seq data.

We also obtained six replicates of control rat data and six replicates of COPD rat data treated with and six replicates of COPD rat data without treated with ECC-BYF III from one of our studies. Briefly, for each group, six Sprague–Dawley rats were included (three males and three females). All the rats weighed 200 ± 20 g, were aged 3 months, and were purchased from Jinan Pengyue Experimental Animal Breeding Co., Ltd. (animal permit number: 1107261911000081; production license no. SCXK[Lu]—20140007). Before the formal experiment, 7 days was needed for the rats to adapt to the environment. This study was approved by the Experimental Animal Care and Ethics Committee of the First Affiliated Hospital, Henan University of Chinese Medicine. The COPD rat model was established by cigarette smoke exposure and bacterial infection (Li et al., 2012). At weeks 1–8, 0.1 ml of *Klebsiella pneumoniae* solution was dripped into the nasal cavity of rats, with the concentration of 6 × 10^8^ CFU/ml, once every 5 days, combined with cigarette smoke exposure 40 min twice daily; the interval was at least 3 h, and the smoke concentration was higher than 3,000 ppm. In weeks 9–12, cigarette smoke exposure was used alone. The control rats were exposed to fresh air and received 0.1 ml of saline solution every 5 days meanwhile. The total modeling time was 12 weeks. Then, the control and model group rats were both given 0.5% CMC Na (0.5 ml/100 g). The ECC-BYF III group rats were given the ECC-BYF III (5.5 mg/kg/day, 0.5 ml/100 g). Finally, for the six replicate samples of each group, the read counts within each gene were calculated.

For the COPD rat data compared with normal controls, edgeR was applied to identify DEGs. For array- and seq-based data from public databases, significance analysis of microarray (SAM) and Wilcoxon rank-sum tests were used to identify DEGs, respectively. Then, Kyoto Encyclopedia of Genes and Genomes (KEGG) pathway enrichment was performed with the DAVID database (Huang da et al., 2009).

### Quantitative Real-Time Polymerase Chain Reaction Assay

The primers were designed and synthesized by GENEWIZ Biotech Co. Ltd. (Suzhou, China) and are shown in Table 2. The total RNA was extracted using a QIAzol^®^ Lysis Reagent (QIAGEN, USA) according to the manufacturer’s instructions. Reverse transcription (RT) was performed using a HiScript^®^ Ⅱ Q RT SuperMix for qPCR (Vazyme, Nanjing, China). The reactions were performed using an QuantStudio 6 real-time fluorescence quantitative PCR System (Life Technologies, Singapore), and the reaction conditions were as follows: 95°C for 30 s as an pre-denaturation step; followed by 40 cycles, each consisting of 95°C for 10 s and 60°C for 30 s; and 95°C for 15 s, 60°C for 1 min, 95°C for 15 s. The values of the target genes were normalized using the value of the housekeeping gene GAPDH through 2^−ΔΔCT^ method. Each group has five samples, and the average values were calculated. Notably, all the mRNA levels are presented as means ± SD. One-way ANOVA test using SPSS 22.0 (IBM Corporation, Armonk, NY, USA) was performed for the statistical analysis.

**TABLE 2:** Primers of reverse transcription PCR analysis for genes.

Gene	Primer	Primer Sequence (5′–3′)	Production length (bp)
*CCR3*	Forward primer	CCT​GCT​GAC​AAT​CGA​CAG​GTA	120
	Reverse primer	GCT​GCC​AAT​ACT​GCA​AGA​CC	
*CCL2*	Forward primer	TAG​CAT​CCA​CGT​GCT​GTC​TC	94
	Reverse primer	CAG​CCG​ACT​CAT​TGG​GAT​CA	
*COL4A2*	Forward primer	CGA​GAG​GCG​TCT​CTG​GAT​TC	200
	Reverse primer	TGC​GTA​AGG​TTC​GCC​TTT​CT	
*VWF*	Forward primer	CCT​TGT​GAA​GTG​GCT​CGT​CT	88
	Reverse primer	GCA​AGT​TGC​AGT​TGA​CCA​GG	
*IL1RN*	Forward primer	ATG​GAA​ATC​TGC​AGG​GGA​CC	78
	Reverse primer	GCC​AGC​TGA​CTC​TGA​ACG​AA	
*IL2RA*	Forward primer	CCA​TAG​TAC​CCG​GCT​GTT​GG	91
	Reverse primer	CCG​TTC​TTG​TAG​GAG​AGG​GC	
*CCL13*	Forward primer	TCA​GTG​TTC​ACC​CCA​GTC​AC	101
	Reverse primer	GGA​CAC​TGG​CTG​CTT​GTG​AT	
*GAPDH*	Forward primer	ACA​GCA​ACA​GGG​TGG​TGG​AC	252
	Reverse primer	TTT​GAG​GGT​GCA​GCG​AAC​TT	

### Protein–Protein Interaction Network Analysis

The PPI network was constructed based on the relevant genes from the STRING database (Szklarczyk et al., 2019) with a combined score greater than 0.4, which is the default score that indicated a medium confidence. Then, the interaction information was downloaded, and the Cytoscape software (v3.8.0) (Shannon et al., 2003) was used to visualize the PPI network. The MCODE plugin (Bader and Hogue, 2003) within Cytoscape was used to identify significant gene clusters and obtain cluster scores with the default parameters (degree cutoff = 2; node score cutoff = 0.2; k-core = 2; max depth = 100). CytoHubba was used to identify hub genes in this network (Chin et al., 2014). For each of the five algorithms (Degree, MCC, MNC, DMNC, and Clustering Coefficient) in the cytoHubba plugin within Cytoscape, we identified the top 30 genes as the hub genes (Cheng et al., 2021). When a gene was identified as a hub gene in at least four algorithms, this gene was considered as the final hub genes.

### Prediction of Target MicroRNAs

Four online miRNA databases (including DIANA-micro T, miRWalk, miRDB, and TargetScan) were used to predict the target miRNAs of genes of interest with the default parameters. In this study, if a gene (such as gene A) could be targeted by one miRNA in at least three of the four databases, this miRNA was defined as the target miRNA of gene A. Then, the co-expressed network of mRNA–miRNA was constructed by Cytoscape.

### Construction of Competitive Endogenous RNA Networks

starBase (version 3.0) was used to predict lncRNAs that interacted with the miRNAs of interest (Li et al., 2014). The screening criteria were as follows: mammalian, human h19 genome, strict stringency (≥5) of CLIP-Data, and with or without degradome data, selected according to a previous ceRNA network study (Cheng et al., 2021). The ceRNA networks of mRNAs, miRNAs, and lncRNAs were constructed using Cytoscape.

## Results

### Differentially Expressed Genes

**FIGURE 1:**
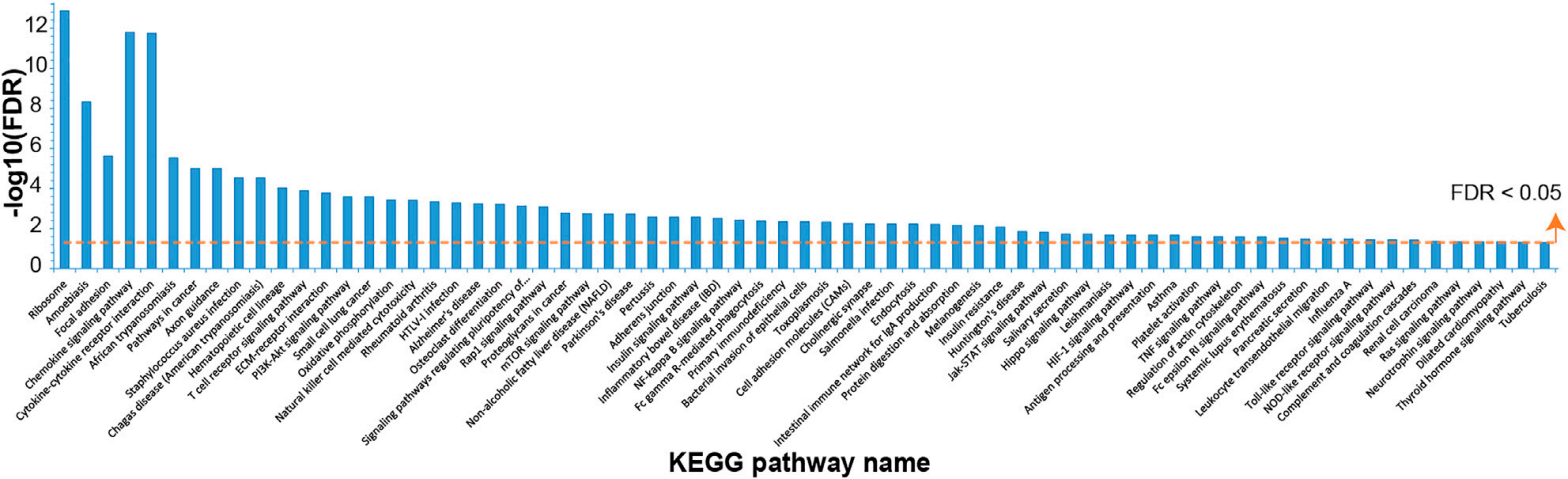
The Kyoto Encyclopedia of Genes and Genomes (KEGG) pathways enriched with dysregulated genes in chronic obstructive pulmonary disease (COPD) rat data compared with control data.

For the gene expression data from six COPD rats in comparison with data from six controls, 3,387 DEGs (1,699 up and 1,688 down) were identified with edgeR (false discovery rate (FDR) < 0.05). A total of 69 significantly disturbed pathways were enriched using the DAVID database with FDR < 0.05, as shown in Figure 1 and Supplementary Table S1. Some of these pathways were reported to be related to COPD, such as the mTOR signaling pathway (Houssaini et al., 2018), NF-kappa B signaling pathway (Wu et al., 2020), PI3K–Akt signaling pathway (Zhang et al., 2020a), and oxidative phosphorylation (Kim et al., 2015), demonstrating the reliability of the identified DEGs.

For the 23 human COPD and nine normal lung tissues from GSE38974, 9,558 DEGs were identified with the SAM algorithm (FDR < 0.2). Similarly, for human COPD and normal lung tissue from GSE8581 (16 COPD and 19 normal) and GSE57148 (98 COPD and 91 normal), 4,347 and 10,009 DEGs were identified, respectively. We evaluated the consistency of the DEGs in each of the two-dataset combinations from the above three datasets. We found the consistency of the DEGs from GSE57148 with each of the other datasets (GSE38974 and GSE8581) was both less than 55%. Thus, the data of GSE57148 were discarded in subsequent analyses. The DEGs from datasets GSE38974 and GSE8581 were highly reproducible, and the consistency ratio was 75.64%, far greater than chance (binomial test, *p* < 1.00E−16). Thus, the 1,304 consistently detected DEGs from the above two datasets were considered as the human COPD-related genes for subsequent analysis.

We then evaluated the consistency of DEGs from human and rat data. First, for the 3,387 DEGs identified from rat data, the Ensemble IDs were ortholog converted to human Entrez Gene IDs using the bioDBnet: dbOrtho database for the consistency analysis. Finally, 2,787 DEGs (1,262 up and 1,526 down) were obtained. For the DEGs from human and rat data, 240 genes were commonly detected, of which 82.5% (198 genes) had an identical dysregulation direction, which could not occur by chance (binomial test *p* = 2.13E−07). Finally, the 198 genes were used for the subsequent analysis.

### Protein–Protein Interaction Network Analysis, MCODE Cluster Modules, and Hub Gene Identification

**FIGURE 2:**
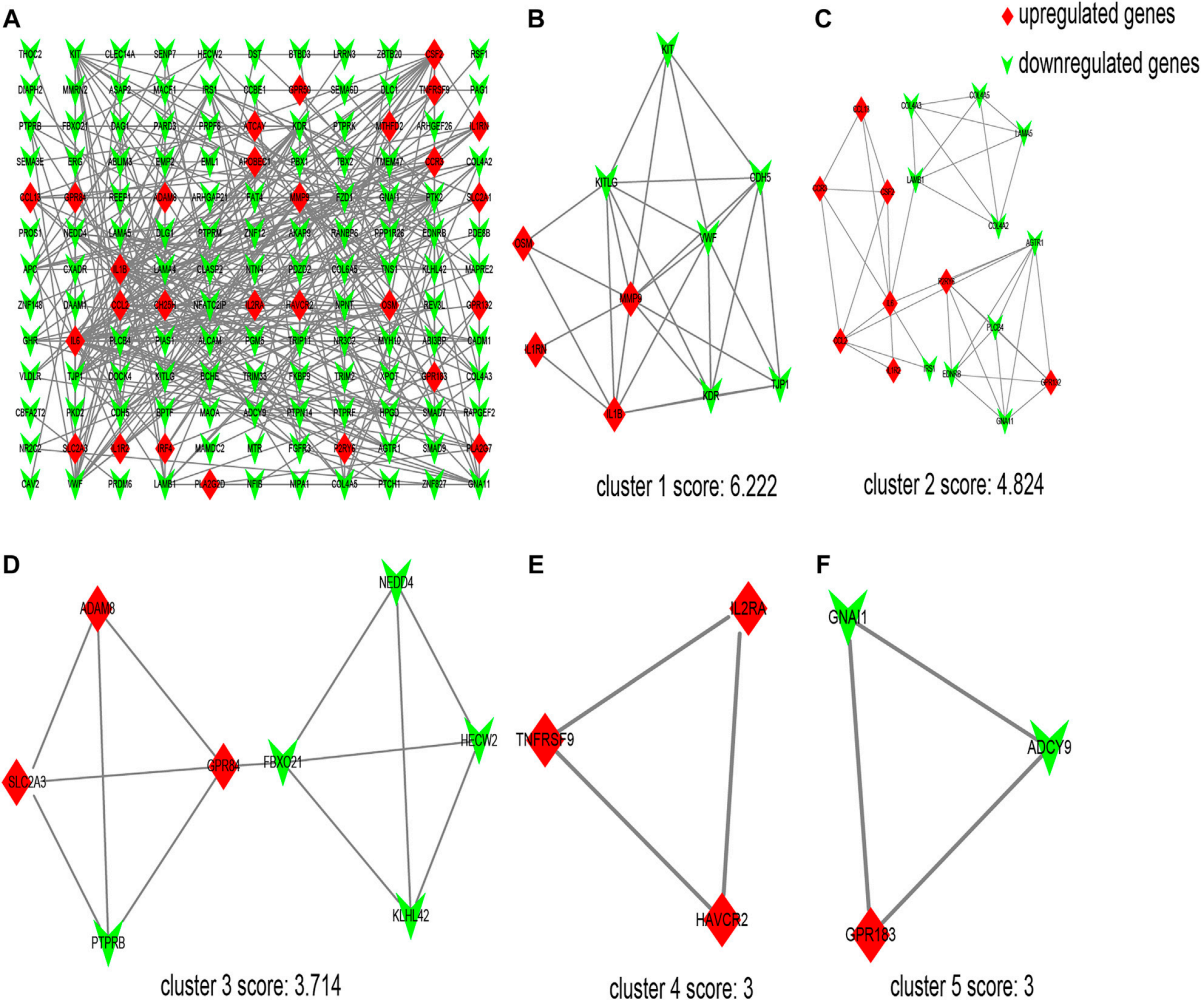
Protein–protein interaction (PPI) network of differentially expressed genes (DEGs) and five cluster modules extracted by MCODE. **(A)** The interaction network between proteins coded by DEGs comprised 144 nodes and 355 edges. Red diamonds represent the upregulated genes, and green V represents the downregulated genes. Five cluster modules extracted by MCODE. Cluster 1 **(B)** had the highest cluster score (score: 6.222, 10 nodes and 28 edges), followed by cluster 2 **(C)** (score: 4.824, 18 nodes and 41 edges), cluster 3 **(D)** (score: 3.714, 8 nodes and 13 edges), cluster 4 **(E)** (score: 3, 3 nodes and 3 edges), and cluster 5 **(F)** (score: 3, 3 nodes and 3 edges).

The interaction network comprising 144 nodes and 355 edges was constructed by STRING and visualized by Cytoscape (Figure 2A), based on the 198 DEGs. In this network, we identified five modules with the MCODE plugin, shown in Figures 2B–F, according to the filter criteria. Cluster 1 had the highest cluster score (score: 6.222, 10 nodes and 28 edges), followed by cluster 2 (score: 4.824, 18 nodes and 41 edges), cluster 3 (score: 3.714, 8 nodes and 13 edges), cluster 4 (score: 3, 3 nodes and 3 edges), and cluster 5 (score: 3, 3 nodes and 3 edges). Next, the cytoHubba plugin within Cytoscape was applied to identify hub genes. For each algorithm (Degree, MCC, MNC, DMNC, and Clustering Coefficient), the top 30 genes were identified, and the results are shown in Supplementary Tables S2–S6. We found that 12 genes were reproducible at least four algorithms (as shown in Table 3).

**TABLE 3:** Frequency of genes that were reproducible among five algorithms.

Gene symbol	Freq
*CCR3*	5
*COL4A2*	5
*COL4A3*	5
*COL4A5*	5
*IL1RN*	5
*IL2RA*	5
*LAMA5*	5
*OSM*	5
*CCL13*	4
*CCL2*	4
*LAMB1*	4
*VWF*	4

Note. Freq denotes the number of genes that were reproducible among the top 30 genes identified by the five algorithms.

### Identification of Interest Genes and Target MicroRNA Prediction

**FIGURE 3:**
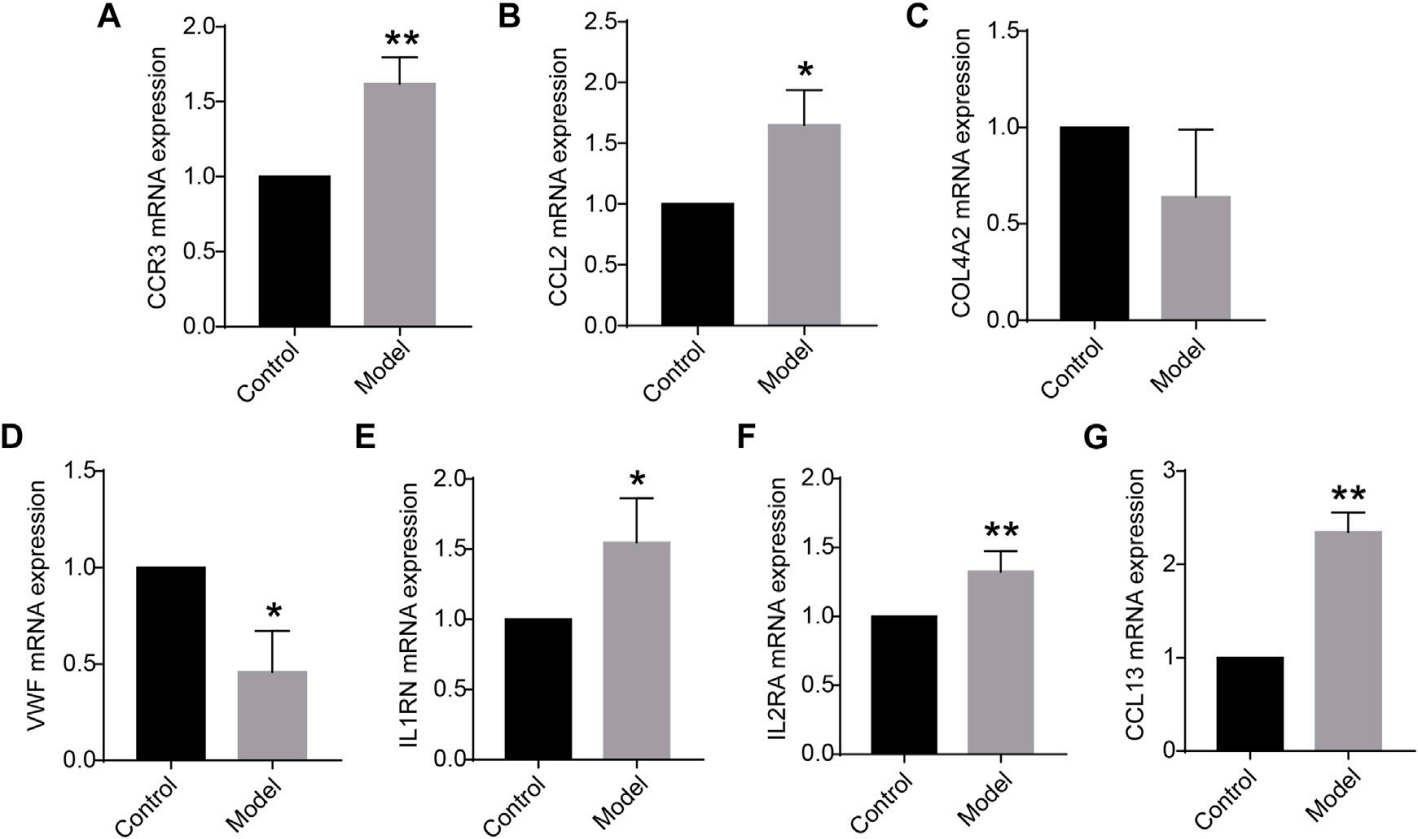
**(A–G)** Messenger RNA (mRNA) expression levels of *CCR3*, *CCL2*, *COL4A2*, *VWF*, *IL1RN*, *IL2RA*, and *CCL13* in the lung tissue (*n* = 5 for each group) vs. control. Data are expressed as mean with SD. *p < 0.05 and **p < 0.01 vs. the control group.

Of the 12 hub genes, we found that *COL4A2* and *VWF* were specifically expressed in lung tissues through the BioGPS database. *CCR3*, *IL1RN*, *IL2RA*, *CCL13*, and *CCL2* were reported to be related to immunity or inflammation, according to the GeneCards database. Moreover, using the rat lung tissues from our laboratory, the mRNA expression levels of *CCR3*, *CCL2*, *COL4A2*, *VWF*, *IL1RN*, *IL2RA*, and *CCL13* were detected by quantitative real-time PCR (qRT-PCR). We found that the mRNA expression levels of *CCR3*, *IL2RA*, and *CCL13* were significantly increased in COPD group compared with those in the control group (*p* < 0.01). The mRNA expression levels of *CCL2*, *VWF*, and *IL1RN* were significantly increased or decreased in the above groups (*p* < 0.05), as shown in Figure 3. It is inspiring that the dysregulation direction of these six genes was consistent with the training data. Moreover, for gene *COL4A2*, though there was no significant difference between the COPD group and the control group (*p* = 0.194), its dysregulation direction was also consistent with the training data. The above results indicated the seven identified genes were reliable. Thus, *CCR3*, *CCL2*, *COL4A2*, *VWF*, *IL1RN*, *IL2RA*, and *CCL13* were selected for further study.

**FIGURE 4:**
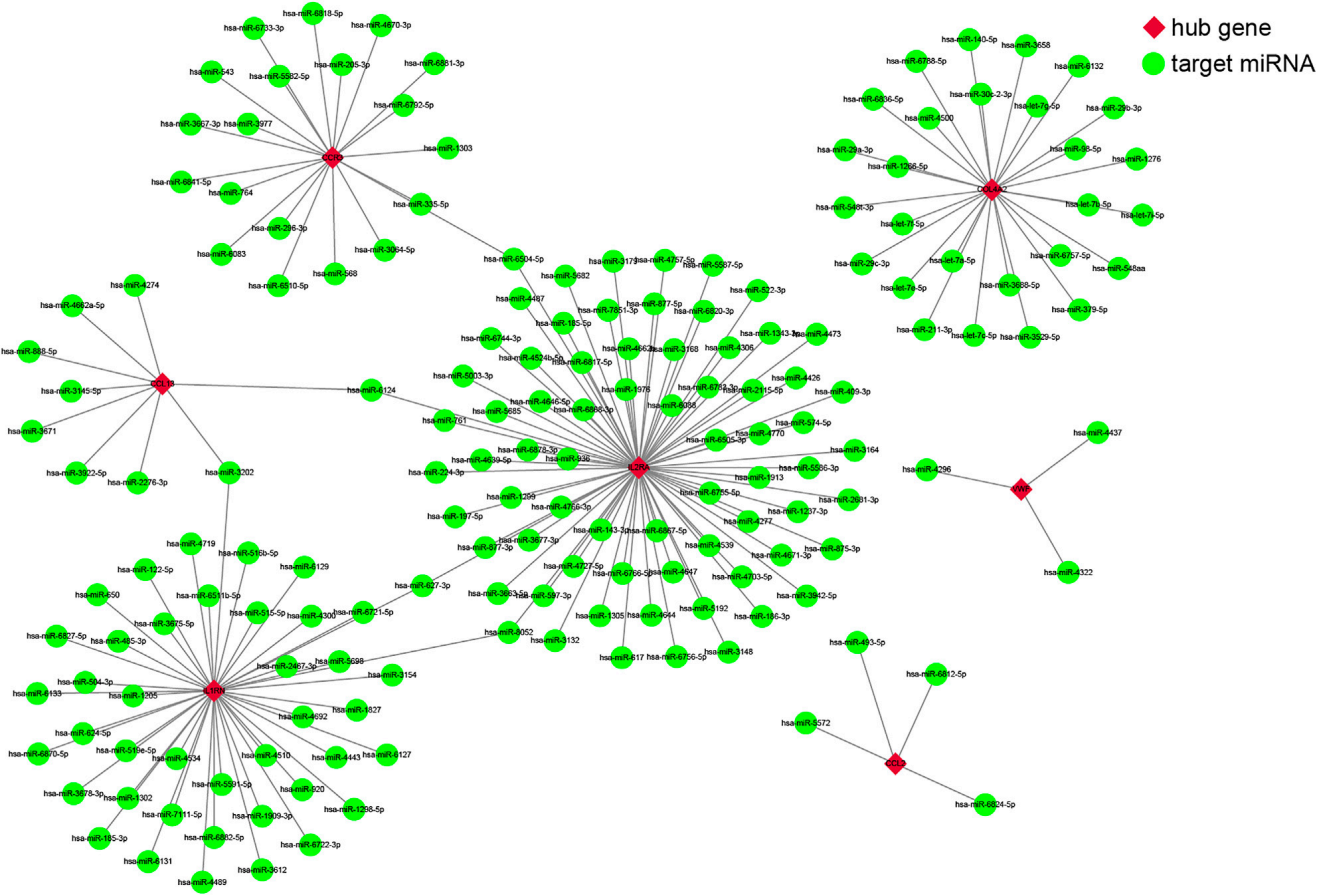
The co-expressed network of messenger RNAs (mRNAs) and target microRNAs (miRNAs). The mRNA-miRNA co-expressed network was constructed by Cytoscape, including 180 nodes and 178 edges. Red diamonds represent the hub genes, and green circles represent miRNAs.

We used four online miRNA databases to predict the target miRNAs of genes of interest. For each gene, the targeted miRNA of the four miRNA databases was obtained, as shown in Supplementary Tables S7–S13. When one particular miRNA could target a gene of interest in at least three databases, the miRNA was defined as the target of this gene. Finally, 173 target miRNAs of seven genes and 178 mRNA–miRNA pairs were obtained, as shown in Supplementary Table S14. The interaction network of mRNAs and miRNAs, comprising 180 nodes and 178 edges, was constructed by Cytoscape, as shown in Figure 4.

### Optimization Verification of the Seven Genes by One Gene Expression Omnibus Dataset and Rat Data

For the 111 COPD and 40 normal lung tissues from GSE76925, the Wilcoxon rank-sum test was applied to evaluate the consistency of the genes of interest. Of the seven genes, the dysregulation direction of *COL4A2*, *VWF*, and *IL1RN* was consistent with the training data (Wilcoxon rank-sum test, *p* < 0.05). For the data from the six COPD rat models and six control rats, those three genes had identical dysregulation directions. Moreover, for the rat COPD model data after ECC-BYF III treatment, the dysregulation direction of the genes was reversed (as shown in Table 4), indicating they might play a key role in traditional Chinese medicine treatments.

**TABLE 4:** The dysregulation direction of *COL4A2*, *VWF*, and *IL1RN* in rat COPD data after ECC-BYF III treatment.

Human_gene	Rat_Ensemble	D (M_vs_C)	D (BYFⅢ_vs_M)	*P* (BYFⅢ_vs_M)
*COL4A2*	ENSRNOG00000023972	DOWN	UP	4.81E-05
*VWF*	ENSRNOG00000019689	DOWN	UP	0.022122494
*IL1RN*	ENSRNOG00000005871	UP	DOWN	0.00109342

Note. D (M_vs_C) denotes the dysregulation direction of genes in rat COPD model compared with rat control data; D (BYFⅢ_vs_M) denotes the dysregulation direction of genes in rat COPD model after ECC-BYF Ⅲ treatment compared with COPD model data without treatment; *P* (BYFⅢ_vs_M) denotes the *p-*value between rat COPD model data with and without ECC-BYF Ⅲ treatment by edgeR method.

### Construction of Competitive Endogenous RNA Networks

**FIGURE 5:**
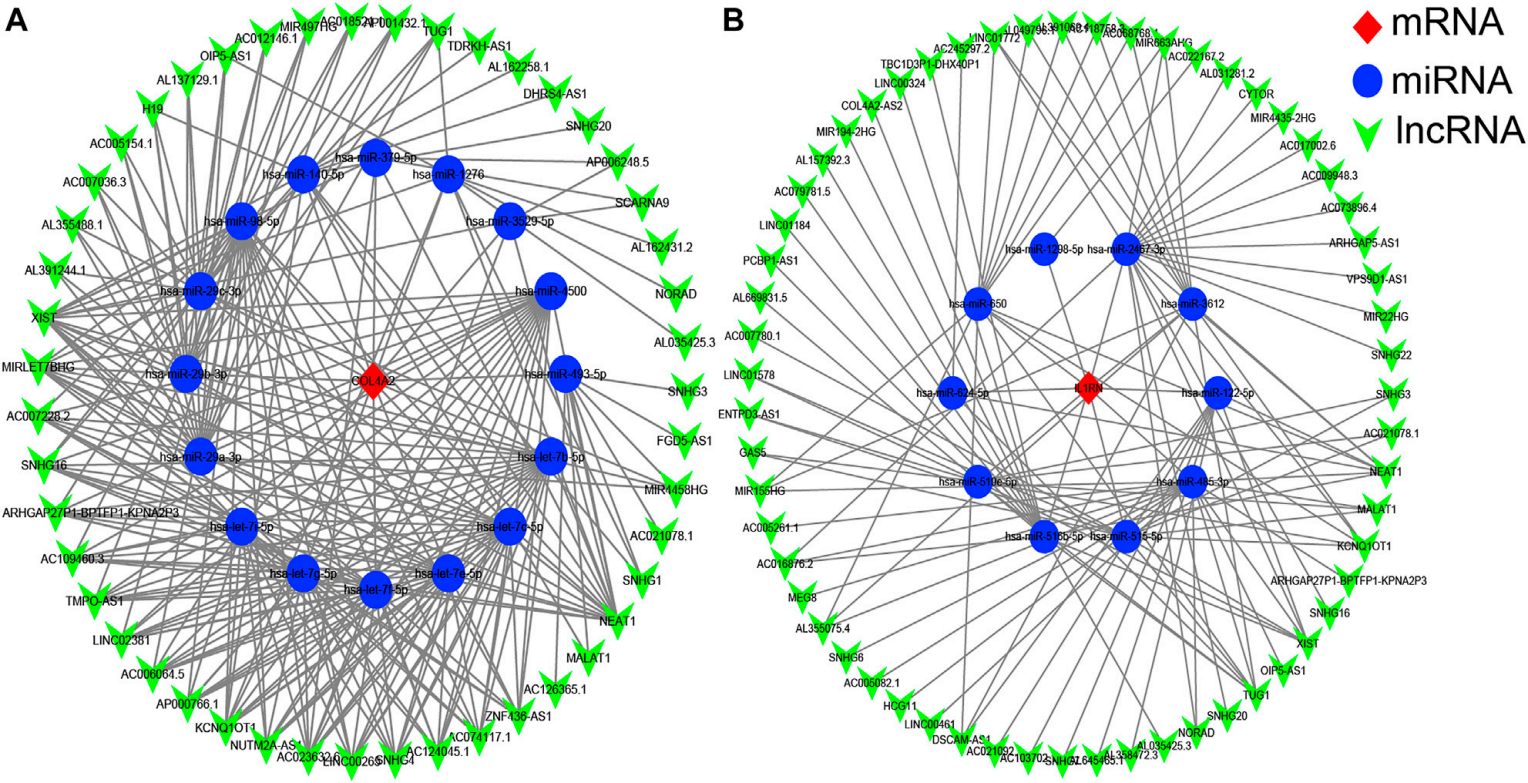
The competitive endogenous RNA (ceRNA) networks of *COL4A2* **(A)** and *IL1RN* **(B)**. Red diamonds represent the hub genes, blue circles represent microRNAs (miRNAs), and green V represents long noncoding RNA (lncRNA).

It is well known that miRNAs can bind mRNAs and induce gene silencing, which further contributes to the decline in the expression level of the gene. In contrast, lncRNAs could combine miRNA response elements and increase the expression level of the corresponding gene. This interaction between RNAs is called a ceRNA network (Salmena et al., 2011). We used the starBase 3.0 database to predict the lncRNAs that interact with the selected miRNAs, and the results are shown in Figure 5. No lncRNA could be predicted for the selected miRNA of *VWF* gene.

## Discussion

In this study, we obtained the DEGs in COPD compared with normal lung tissues using the multiple gene expression data from human and rat tissues. Then, based on PPI networks and the five cytoHubba algorithms, 12 hub genes were identified. From these, we further selected the genes (*CCR3*, *CCL2*, *COL4A2*, *VWF*, *IL1RN*, *IL2RA*, and *CCL13*) related to immunity/inflammation or specifically expressed in lung tissues through the BioGPS and GeneCards databases, and they were validated by qRT-PCR. Then, we predicted the targeted miRNA of those genes. The genes were further verified and optimized using a GEO dataset and COPD rat data. We found that *COL4A2*, *VWF*, and *IL1RN* were consistent with all the training data, GEO verification data, and rat data; and the dysregulation direction of the three genes was reversed when treated with ECC-BYF III, providing direction for COPD treatment. Of the genes, *VWF* and *IL1RN* were reported to be related to COPD. Studies show that *VWF* is a signature of inflammation in COPD, and its levels might reflect the persistence of chronic inflammation in COPD (Langholm et al., 2020). The polymorphisms of *IL1RN* are implicated in the pathogenesis of COPD and constitute a risk factor for COPD occurrence in East Asians (Xie et al., 2014). In addition, we constructed the ceRNA networks, providing a novel approach to explore the pathogenesis of COPD at the transcriptome level.

COPD is a common respiratory disease that severely compromises patients’ quality of life and imposes heavy economic burdens on patients, families, and society (Ma et al., 2019; Mao et al., 2019). Although several medicines, including inhaled corticosteroids, bronchodilators, and long-acting β2-agonists, have already been shown to have some clinical efficacy, they also have many adverse effects and are time- or dose-dependent (Cazzola et al., 2019). For example, patients treated with β2 receptor agonists may suffer from rapid heartbeat, muscle tremors, and metabolic disorders (Billington et al., 2017), while those taking anticholinergic drugs may suffer from dry mouth, blurred vision, and cardiac rhythm disturbances (Tune, 2001). Traditional Chinese medicine has some advantages in the treatment of COPD. Significant clinical efficacy and few side effects were reported for ECC-BYF III treatment of COPD (CN. 201811115372.3). Thus, further exploration of traditional Chinese medicine as a treatment approach for COPD is warranted.

In this study, we applied a discovery-driven analysis to identify DEGs and found three genes of interest (*COL4A2*, *VWF*, and *IL1RN*) by constructing a PPI network and were verified in one GEO dataset of human and rat data from our laboratory. The genes were reversed when the COPD rat model was treated with ECC-BYF III. The mechanism of these genes in COPD deserved to be explored in the further study.

This study also has some limitations. For the DEG analysis of the COPD data from public database, a relatively lenient threshold value (FDR < 0.2) was used because no DEG could be identified in GSE8581 with the stricter threshold (FDR < 0.05 or even FDR < 0.1). Fortunately, in the DEGs from the two datasets (GSE38974 and GSE8581) with FDR < 0.2, we found that 1,724 genes were common, and 75.6% of genes (1,304) have the identical dysregulation direction, which would not occur by chance (binomial test, *p* < 1.00E−16). These results demonstrate that the DEGs identified under the threshold of 0.2, were reliable, and the cutoff used was logical. In the future, with the accumulation of COPD data from public databases, more COPD tissue samples, or an improved algorithm should be considered. In addition, our study focused only on lung tissue. Thus, we also searched alveolar macrophages and peripheral blood mononuclear cell (PBMC) data for similar analyses. For the 22 human COPD and 66 normal alveolar macrophages from GSE38974, 9,268 DEGs were identified with the SAM algorithm (FDR < 0.2). Similarly, for human COPD and normal alveolar macrophages from GSE13896 (12 COPD and 58 normal), 7,332 DEGs were identified. From the two lists of DEGs identified, 6,284 genes were commonly identified, and 99.98% (6283 genes) have an identical dysregulation pattern, unlikely by chance (binomial test, *p* < 1.00E−16), proving the robustness of the identified DEGs. Therefore, the 6,283 consistently detected DEGs from the above two datasets were considered the human COPD alveolar macrophage-related genes for subsequent analysis. As for the 1,304 human COPD-related genes identified from lung tissue, we first evaluated the consistency of the DEGs from lung tissues and alveolar macrophages. Of the 1,304 and 6,283 DEGs identified, 348 genes were commonly identified, and 56.32% (196 genes) had the same dysregulation pattern, with weak statistical significance. These results indicate a large heterogeneity between lung tissues and alveolar macrophages. Considering the small number of genes of interest, KEGG pathway enrichment analysis was performed with a relatively lenient threshold (*p* < 0.1, default parameter in the DAVID database). For the 196 DEGs consistently identified in the two types of tissue, 11 pathways were enriched, as shown in Supplementary Table S15, indicating that these pathways might participate in COPD pathogenesis in both alveolar macrophages and lung tissue. Some of the pathways, such as extracellular matrix (ECM)–receptor interaction (Zhang et al., 2020b), Epstein–Barr virus infection (McManus et al., 2008), and PI3K–Akt signaling pathway (Zhang et al., 2020a), were reported to be linked to COPD. As for the 152 genes commonly identified but with a reversed dysregulation pattern, four pathways were enriched, as shown in Supplementary Table S16, indicating that these pathways might also participate in COPD pathogenesis but with different patterns in alveolar macrophages and lung tissue. Some of these pathways such as the Jak-STAT signaling pathway (Zhao et al., 2020) and cytokine–cytokine receptor interaction (Zhao et al., 2018) were reported to be related to COPD. As for the PBMC sample of COPD individuals, only one dataset GSE42057, including 94 COPD and 42 control samples, could be found. Only 167 DEGs could be identified (SAM, FDR < 0.2), possibly due to the complex signals of blood itself or the sample quality. To compromise, we also evaluated the consistency of the DEGs from lung tissues and PBMC for the 1,304 and 167 DEGs identified from them. A total of 19 genes were commonly identified, and 94.74% (18 genes) had identical dysregulation, unattributable to chance (binomial test, *p* = 3.81E−05). These results indicate that there are common features between lung tissues and PBMC. As COPD alveolar macrophage and PBMC data accumulate, the commonality and specificity of COPD pathogenesis in alveolar macrophages, PBMC, and lung tissue should be further investigated.

Moreover, in this study, the cutoff of 0.4, which did not discriminate the interaction of experimentally validated or predicted data, was used to construct the network. The obtained network might not have high confidence. Thus, we also constructed the PPI network using the cutoff of 0.4 and interaction sources from experimentally validated data. Based on the 198 DEGs identified in this study, the interaction network comprising only 35 nodes and 23 edges was obtained, as shown in Supplementary Figure S1. Though the interaction between proteins has high confidence, they lose much interaction information, which prevents us from performing the subsequent network analysis, including the hub gene identification with cytoHubba. Fortunately, the result of the network analysis in this study was further optimized and well validated in BioGPS, GeneCards databases, independent data from public databases, and qRT-PCR data from our laboratory. The above multistep verification could circumvent the issues of not high confidence for interaction networks, and the final obtained key genes were reliable. Additionally, 0.4 is a commonly used cutoff in PPI network analysis (Li et al., 2021; Yan et al., 2021; Zhang et al., 2021). In summary, the cutoff of 0.4, even without discriminating the interaction of experimentally validated or predicted data, is a relatively reasonable selection in this study.

## Data Availability

The datasets presented in this study can be found in online repositories. The names of the repository/repositories and accession number(s) can be found in the article/Supplementary Material.

## Supplementary Material

Supplementary Figure S1 The protein-protein interaction network comprised 35 nodes and 23 edges with the cutoff of 0.4 and interaction sources from experimentally validated.
