# Supplementary figures and images for: Identification of Potential Key Genes in the Pathogenesis of Chronic Obstructive Pulmonary Disease Through Bioinformatics Analysis
Source: Front Genet. 2021 Nov 3;12:754569. doi: 10.3389/fgene.2021.754569 (PMC8595135; doi:10.3389/fgene.2021.754569)

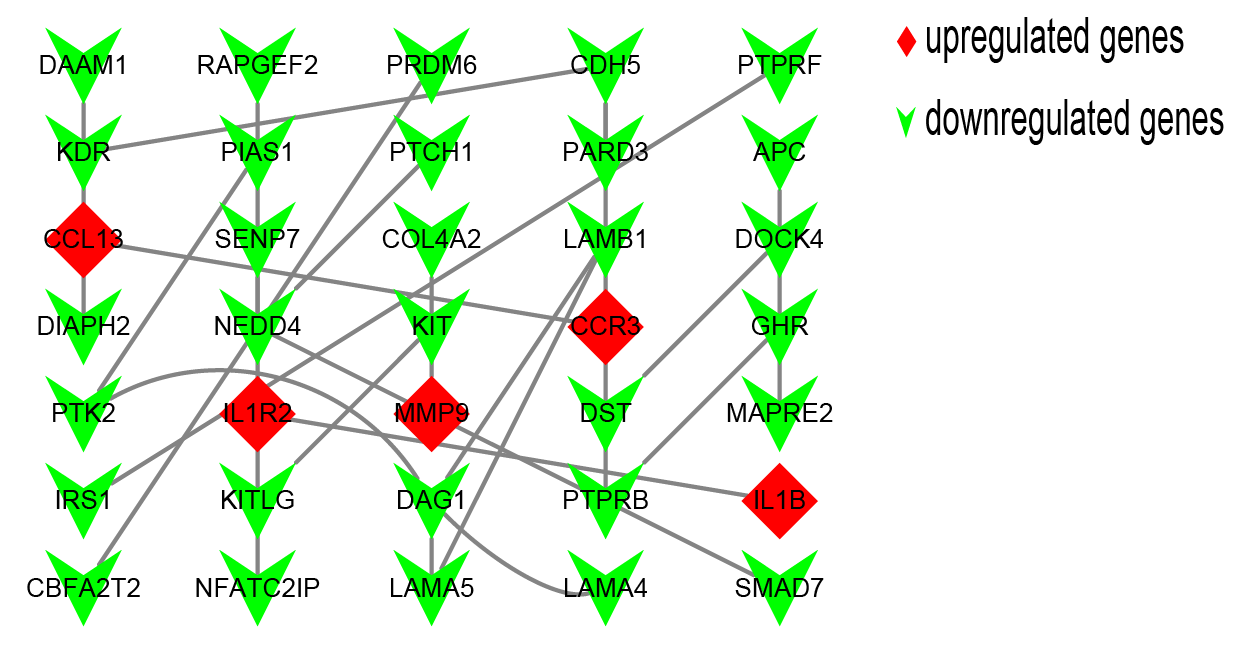

Supplement: Supplementary file 1 [file Image1.TIF]
